# The Modified Iliopubic Tract Repair—A Pain-Free Alternative

**DOI:** 10.1055/s-0038-1653980

**Published:** 2018-05-23

**Authors:** Iqbal Ali, Vashisht Dikshit, Kshitij Manerikar, Mirat Dholakia, Maitreyee Save

**Affiliations:** 1 Department of Surgery, Dr. D. Y. Patil Hospital, Dr. D. Y. Patil Vidyapeeth, Pimpri, Pune

**Keywords:** hernia, inguinal, herniorrhaphy, mesh, chronic pain, pain, postoperative

## Abstract

**Background** The open preperitoneal repair offers the benefits of placing the mesh in the preferred position while avoiding the disadvantages of laparoscopic repair.

**Methods** A total of 60 patients with bilateral inguinal hernias were randomized to undergo either the standard Lichtenstein meshplasty or the modified iliopubic tract repair in a teaching hospital. Outcomes measured were immediate postoperative pain, return to activity, and delayed neurological complications.

**Results** Patients who underwent the iliopubic tract repair walked out of bed faster than the Lichtenstein group (6.3 hours vs 7.4 hours, *p* < 0.0001) and experienced significant lower pain as charted by visual analogue scale scores (3.28 vs 2.71 on day 1, 2.16 vs 1.71 on day 2, 1.92 vs 1.08 on day 3; *p* < 0.05). Delayed complications like chronic inguinal pain and numbness were not seen in the iliopubic tract group. However, this difference was not statistically significant ( *p* > 0.05).

**Conclusion** The iliopubic tract repair offers an excellent alternative to the Lichtenstein meshplasty, and is associated with lower postoperative pain, earlier return to work, and lower delayed neurological complications.

A few surgical conditions can boast of such a multitude of management options as inguinal hernias. The surgeon of today has a vast range of surgical techniques at his disposal, ranging from anatomical repairs to the modern laparoscopic repairs. Among these, the modified Lichtenstein meshplasty remains the most popular. This popularity can be attributed to its ease of learning, safety, and low recurrence rates. 1

This technique is not without its drawbacks. Postoperative pain, numbness, and chronic groin pain continue to plague patients following meshplasty. The former can be attributed to the greater number of nerves encountered during the anterior approach used in the Lichtenstein technique. 2 These complications are magnified in cases of bilateral and recurrent inguinal hernias. The difficult anatomy encountered in cases of recurrent hernias raises the need for extensive and complicated dissection, which consequently increases rates of postoperative complications. This highlights the need to consider other surgical techniques when faced with bilateral or recurrent inguinal hernias.

The open preperitoneal approach has the advantage of placing the mesh in the preferred location, namely, the preperitoneal space, while eschewing the problems associated with laparoscopy. 2 This approach also minimizes dissection in the inguinal canal, resulting in lesser manipulation of inguinal nerves and potential damage to the vital structures, as well as the other complications of operating in a nonvirgin field. 2

This study compared a modification of the iliopubic tract repair, wherein a single midline incision is used for bilateral repair, to the standard Lichtenstein meshplasty with respect to postoperative pain, time taken to return to work, and neurological complications (chronic groin pain and numbness).

## Material and Methods

A prospective randomized study of 60 patients was performed at our hospital between May 2015 and May 2017. The study protocol followed the guidelines stated by the CONSORT criteria. The sample size was calculated using the formula:

*n* = *z* ^2^ x *P* (100– *P* )/ *d* ^2^

Where:

*P* was the anticipated prevalence

*d* was the desired precision

*z* was the appropriate value from the normal distribution for the desired confidence, which was 95% in our study ( *z* = 1.960).

Demographic details of all patients were recorded. Patients between ages 18 and 80, with bilateral uncomplicated inguinal hernias, were randomized into two groups: one undergoing the Lichtenstein meshplasty, and the other, the modified iliopubic tract repair. Patients with unilateral, complicated, congenital, and recurrent hernias were excluded from the study ( Table 1 )

**Table 1:** Patient characteristics

	Lichtenstein	Modified iliopubic
**Mean age**	61.77	61.7
**Type of hernia**		
B/L direct	12	9
B/L indirect	11	10
U/L direct + U/L Indirect	3	6
Pantaloons	4	5

Institute Ethics Committee clearance was obtained prior to initiation of the study. Informed consent was obtained from all patients after explaining the nature of the study, and the advantages and disadvantages associated with both procedures.

All the patients in the study were operated upon by the same team of surgeons comprising experienced consultants, as well as surgery residents.

Both groups received inj. cefotaxime 1 g intravenously (IV) at the time of induction of anesthesia as per our institute protocol.

Patients in Group A underwent the standard Lichtenstein meshplasty repair as described in literature. A 3”x 6” lightweight Prolene mesh was fixed over the posterior wall of the inguinal canal using interrupted Prolene sutures, and the procedure repeated on the opposite side after repair of one side.

**Fig. 1:**
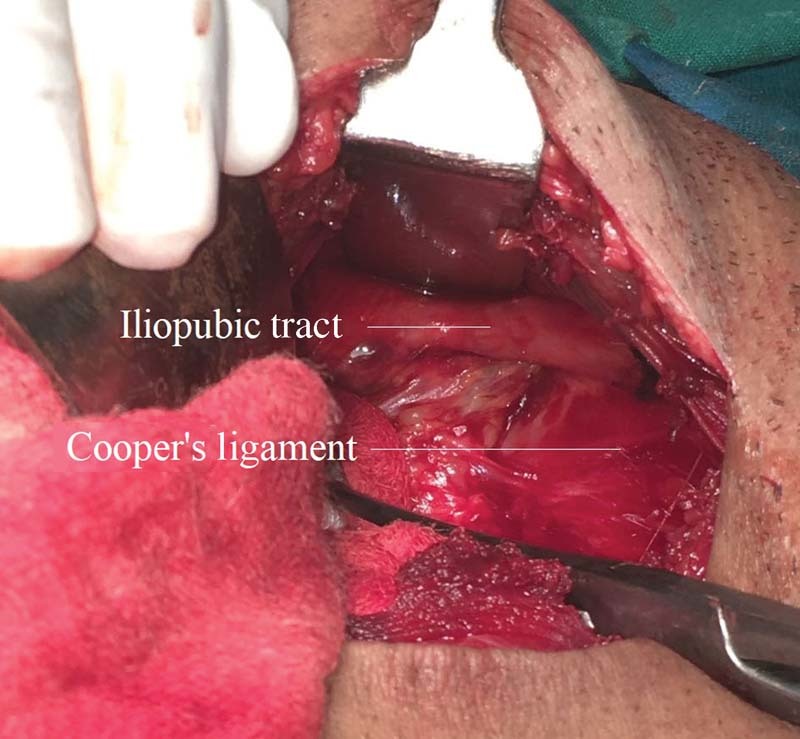
The iliopubic tract.

**Fig. 2:**
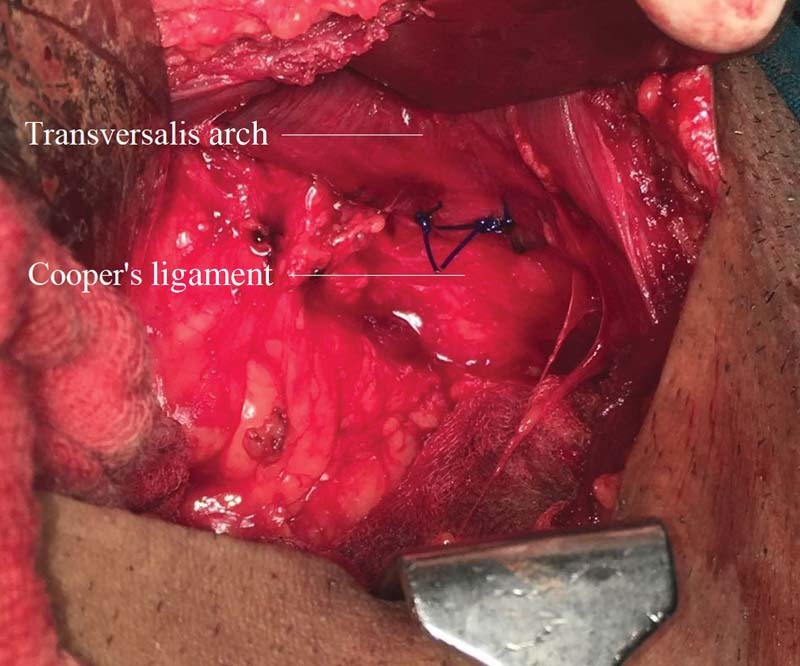
Approximating transversalis arch with iliopubic tract with sutures.

**Fig. 3:**
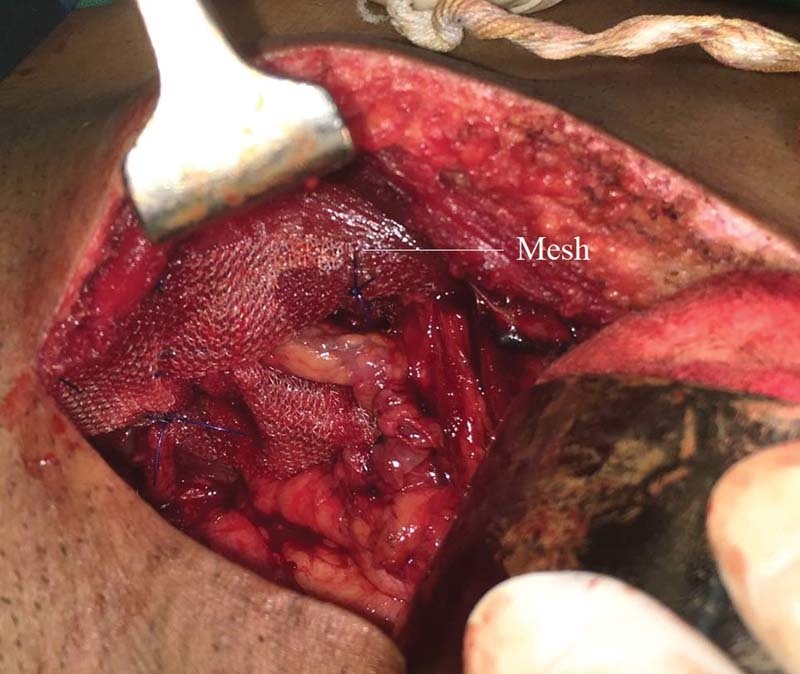
Placement of Prolene mesh over posterior wall in preperitoneal plane.

Patients in Group B underwent the modified iliopubic tract repair, wherein the preperitoneal space was accessed using a lower midline incision, extending from below the umbilicus to the pubic symphysis. The hernia sac was then identified. In case of indirect inguinal hernia, the sac was ligated and divided at the level of the deep ring, with the distal part of the sac remaining within the canal. In case of a direct hernia, the sac was inverted with a running purse-string suture. Repair was then done by approximating the arching fibers of the transversalis fascia superiorly to the iliopubic tract ( Fig. 1 ) inferiorly with interrupted Prolene sutures ( Fig. 2 ). A small mesh (3”x 6” lightweight Prolene mesh cut in half) was then sutured placed over the repair, and secured superiorly to the transversalis arch, and inferiorly to the pectineal ligament, thus eliminating possibility of future femoral hernias as well ( Fig. 3 ). The contralateral hernia was similarly repaired through the same incision. The incision was then closed in layers over a suction drain.

Both groups received inj. cefotaxime 1 g IV 12 hourly for 3 days as per institute protocol for antibiotic prophylaxis, and inj. paracetamol 1 g IV 8 hourly for analgesia postoperatively.

Main outcome assessed was postoperative pain, with time taken to walk out of bed, long-term pain, and numbness being secondary outcomes assessed.

Patients were assessed immediately postoperatively for pain. Pain was charted using the 10-point numerical visual analogue scale (VAS) every 8 hours until discharge. Time taken to walk out of bed was defined as the time taken for the patient to independently stand and walk out of bed.

Patients were additionally followed up 3 monthly, for a period of up to 2 years. They were asked about inguinal pain and history of consuming analgesics. Patient were also subjected to a physical examination to assess numbness and paraesthesia. Follow-up examinations were conducted by a member of the team who was not the operating surgeon for the particular case to avoid bias.

Tabulated VAS scores were assessed for significance using the Mann–Whitney U test. Time taken to walk out of bed, and chronic pain was compared using the unpaired *t* -test. A *p* value less than 0.05 was considered statistically significant.

## Results

**Fig. 4:**
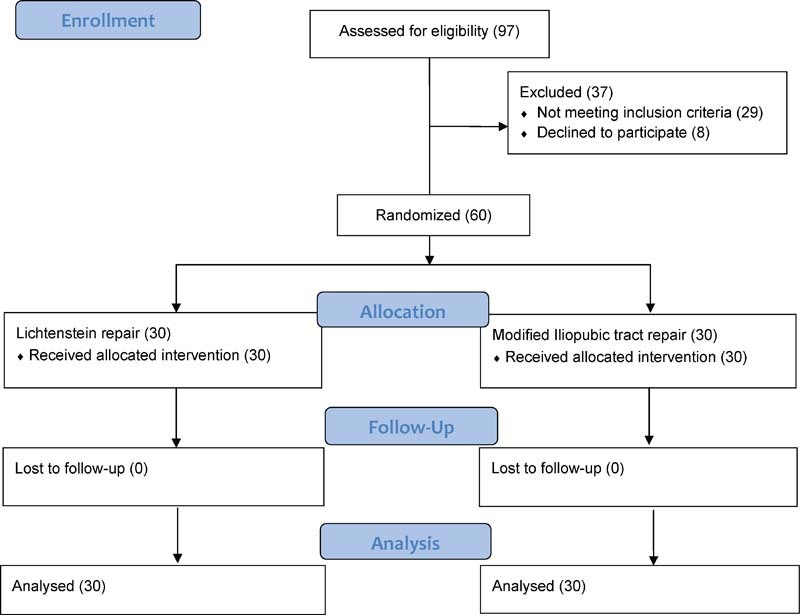
Participant flow diagram.

All 60 (30 in Lichtenstein group, 30 in iliopubic tract group) patients completed the study. None were lost to follow-up ( Fig. 4 ).

Majority of the patients were in the 61 to 70 years bracket (Group A: 36.67%, Group B: 40%). The youngest patient in the study was 24 years old, while the oldest was 80 years old. Both groups were comparable, with no statistical difference in age ( *p* value = 0.49). All patients in the study were males.

Patients in the iliopubic tract repair group walked out of bed faster than those after Lichtenstein repair (6.33 ± 0.488 hours vs 7.4 ± 0.85 hours). This difference was significant at 95% confidence interval ( *p* < 0.0001) ( Table 2 ).

**Table 2:** Time taken to walk out of bed

Group	Time take to walk out of bed (in hours)
Lichtenstein	7.4
Modified iliopubic tract repair	6.33

**Fig. 5:**
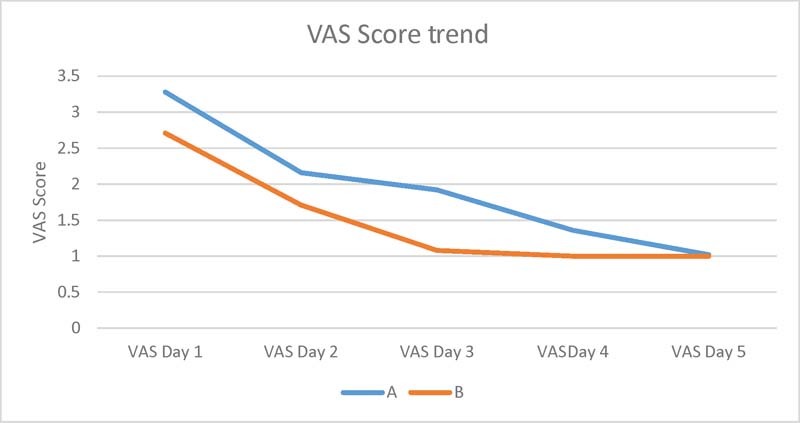
Difference in mean visual analogue score (VAS) scores between both groups.

Patients in the Lichtenstein group had higher VAS pain scores postoperatively compared with the iliopubic tract repair group up to the third post-op day ( *p* < 0.05). Difference in pain was not significant on days 4 and 5 ( *p* > 0.05) ( Fig. 5 )

More patients in the Lichtenstein repair group experienced delayed complications (numbness, chronic pain) as compared with the iliopubic tract repair group. However, this difference was statistically insignificant ( *p* > 0.05) ( Table 3 ).

**Table 3:** Delayed postoperative complications in both groups

	Lichtenstein	Modified iliopubic	*p* value
*Chronic inguinal pain*	1	0	0.8
*Groin numbness*	5	0	0.052

## Discussion

The ideal method of hernia repair would cause minimal discomfort to the patient, both during the surgical procedure and in the postoperative course. It would be technically simple to perform, and easy to learn, would have a low rate of complications and recurrence, and would require only a short period of convalescence.

Most modern studies compare the standard Lichtenstein meshplasty to laparoscopic techniques, and few directly compare it to open preperitoneal methods. This makes it difficult to assess the impact of the posterior approach itself in the surgical outcome, factoring in the minimal trauma caused by laparoscopic methods. The present study design makes it possible to directly study the role of the posterior approach alone in outcome of hernia repair by comparing it to the current gold standard.

The results can vary widely among different centers, and the results from specialized centers are often good. For example, very low recurrence rates have been reported from the Shouldice Hospital using their eponymous technique, even <1%, 3 with some authors suggesting it be used as a gold standard when evaluating new herniorrhaphy techniques. 4 However, there exists a steep learning curve for the technique, 5 which has resulted in other centers failing to reproduce the Shouldice Hospital's stellar recurrence rates. 6 Our study was conducted in a general surgical teaching center, which far better mimics clinical reality.

The Nyhus repair is considered the standard open preperitoneal repair. Our study focused on our modification of the same, tailored specifically to bilateral inguinal hernias. This choice was also relevant as it considered the cost of the repair, which is sensitive in a developing country like ours.

The iliopubic tract repair showed significantly shorter time taken to walk out of bed among patients as compared with the Lichtenstein meshplasty (6.33 hours vs 7.4 hours). Multiple studies also demonstrated the same, clearly giving the open preperitoneal approach the advantage. 7 8 9 10 11 Lower pain in the iliopubic tract group might probably explain the same.

The most significant advantage for the iliopubic tract group was in terms of postoperative pain. Mean VAS scores for the iliopubic tract repair group following surgery were significant lower than those for the Lichtenstein group in the immediate postoperative period, until the third postoperative day (3.28 vs 2.71 on day 1, 2.16 vs 1.81 on day 2, 1.92 vs 1.08 on day 3). Mean VAS scores showed no statistical difference toward the end of day 4 and on day 5.

Postoperative pain following hernia repair has extensively been studied, and most reports show a distinct advantage with the posterior preperitoneal approach, as employed by the iliopubic tract repair. A meta-analysis of over 500 patients showed significantly higher pain following Lichtenstein repair as compared with preperitoneal repair. 7 Other studies by Liu et al, Koning et al, Nienhuijs et al, Ray et al, and Sajid et al also found significantly lower pain scores in patients following open preperitoneal repair as compared with the Lichtenstein meshplasty. 2 8 9 10 12

Late complications are a bane of hernia repair. Our study assessed patients for groin numbness, chronic pain, recurrence, and late infection at 3 monthly intervals for a maximum period of 2 years. Long-term pain was measured based on a history of analgesic use, and restriction of daily activities. While other indices are available, we found their application difficult with our patients, who mostly are illiterate and come from poor backgrounds. Late complications were encountered more in the Lichtenstein group, with five patients reporting numbness, one each reporting chronic groin pain and recurrence. None of the patients who underwent the iliopubic tract repair suffered any delayed complication. However, this was not statistically significant, and a more extensive study would be required to confirm statistical advantage.

Our findings, however, were in contrast to others, who have reported significantly higher rates of groin numbness and chronic inguinal pain among patients undergoing the Lichtenstein meshplasty as compared with preperitoneal repairs. It has been postulated that this may be due to the greater chances of nerve damage in the anterior approach employed by the Lichtenstein repair. 6 7 8 9 11 12 13

## Conclusion

The modified iliopubic tract repair has shown several clinically relevant advantages. Patients experienced significantly lower pain after the iliopubic tract repair and walked out of bed faster as well. They also experienced lower rates of neurological complications. Immediate postoperative complications were on par with the high standards set by the Lichtenstein meshplasty.

For surgeons who prefer an open approach, the modified iliopubic tract repair is a stellar alternative to the Lichtenstein meshplasty, especially for bilateral and recurrent hernias.
